# Tirofiban-Induced Thrombocytopenia Occurring with Crohn's Disease

**DOI:** 10.1155/2016/4605139

**Published:** 2016-04-06

**Authors:** Toni Ibrahim, Fady El Karak, Assem Araji, Elie El Rassy

**Affiliations:** ^1^Department of Cardiology, Bekaa Hospital, Zahle, Lebanon; ^2^Department of Hematology, Hôtel-Dieu de France University Hospital, Faculty of Medicine, Saint Joseph University, Beirut, Lebanon

## Abstract

A 69-year-old man, with severe refractory Crohn's disease, presented with acute coronary syndrome that required angioplasty. He developed severe tirofiban-induced thrombocytopenia (TIT) heralded by type I allergic reaction that required steroids and a combination of antihistamine H1 and antihistamine H2 for symptomatic management. The thrombocytopenia spontaneously resolved uneventfully in 48 hours thereafter. This case report suggests a possible association between TIT and inflammatory bowel disease. Therefore, strict monitoring of the platelet count is required in patients who develop allergic reactions to tirofiban.

## 1. Introduction

Glycoprotein IIb/IIIa (GP IIb/IIIa) receptor is a platelet surface protein that leads to platelet aggregation via cross-linking of fibrinogen and von Willebrand factor. Therefore, it has been considered as a primary target for the treatment of acute coronary syndrome and used as an adjunct to coronary interventions [1]. Although thrombocytopenia occurring with the GP IIb/IIIa inhibitors has been described in only 2% of patients, it constitutes a major safety concern with poor prognosis and high rates of mortality [2]. The pathophysiology of thrombocytopenia in this setting is not yet fully clear with various clinical presentations concerning its time of onset and severity [3]. Herein, we report the case of an early severe tirofiban-induced thrombocytopenia (TIT) heralded by type I allergic reaction.

## 2. Case Report

A 69-year-old man without previous medical history except for a refractory Crohn's disease was referred to the Cardiology Department for the management of an acute coronary syndrome. His labs were normal except for an increased level of cardiac biomarkers. The patient received aspirin 100 mg PO and enoxaparin 30 mg IV before undergoing an urgent coronary angiogram. The angiogram revealed 95% stenosis of a dominant marginal branch requiring stenting. The patient therefore received enoxaparin 25 mg IV, ticagrelor 180 mg PO, and tirofiban 34 mcg/Kg/min followed by 0.1 mcg/kg/min over 24 hours. The rest of the angioplasty was uneventful. One day later, the patient complained of palpitations, dyspnea, chills, and minor urticaria. The patient was symptomatically treated with methylprednisolone 60 mg IV STAT and was started on desloratadine 5 mg PO q12h and ranitidine 150 mg IV q12h. One day later, the patient complained of repetitive episodes of rectal bleeding. The blood exam was only relevant for severe thrombocytopenia at 2000/mm^3^ with normal peripheral blood smear. The patient received two pools of platelets at 12 hours apart. Tirofiban, ticagrelor, enoxaparin, and aspirin were stopped. Two days later, the platelet count increased to 47000/mm^3^. The patient was discharged three days later with normal hematologic test results.

## 3. Discussion

Since Andreas Gruntziq first introduced coronary angioplasty in 1977, clinicians have been driven to lower the risks of stent thrombosis and introduced anticoagulants and antiaggregants to their procedures. Clinical studies have proved a significant benefit for these adjunctions but with a relatively acceptable risk of bleeding and thrombocytopenia [4]. In the particular case of GP IIb/IIIa inhibitors, the platelet GP IIb/IIIa receptor is inhibited and thus fibrinogen binding and platelet/platelet aggregation are blocked. The pathophysiology of thrombocytopenia associated with the use of GP IIb/IIIa receptor inhibitors may be described as acute and severe (<50.000 platelets/mm^3^) within 12 hours of exposure, acute within 12 hours of second exposure, severe or not 5 to 7 days after any type of exposure, and pseudothrombocytopenia [5]. However, it is often possible to find more than one etiology responsible for thrombocytopenia including acute, idiosyncratic, and delayed immune-mediated mechanisms especially with the concomitant use of heparin, ticagrelor, and aspirin [3].

Overall, drug-induced immune thrombocytopenia (DITP) is a form of immune thrombocytopenia (ITP) that occurs 5 to 14 days after initiation of a new drug (commonly quinine or antibiotics). This delay is equivalent to the time for the patient to become sensitized to the drug and to constitute antibodies against his platelets [6]. In contrast, TIT is nearly always caused by naturally occurring drug-dependent antibodies within minutes to hours after the first exposure to the drug. Interestingly, GP IIb/IIIa inhibitor-induced thrombocytopenia is similar to heparin-induced thrombocytopenia (HIT) time pattern occurring within 1–4 hours or 7–14 days [7]. In general, DITP is a clinical diagnosis mainly based on the patient's history of recent drug initiation and the resolution of thrombocytopenia upon drug withdrawal. Testing for drug-dependent anti-platelet antibodies may be helpful in establishing the diagnosis. However, thrombocytopenia may only be recognized days thereafter when bleeding occurs as in our case.

The comparison of the incidence of thrombocytopenia across the different GP IIb/IIIa inhibitors has been recently reported in one meta-analysis of 29 large placebo-controlled randomized trials [2]. Overall, GP IIb/IIIa inhibitors, administered PO or IV, increase the rate of thrombocytopenia (RR = 1.62; 95% CI 1.48–1.78) and severe thrombocytopenia (RR = 3.52; 95% CI 2.87–4.30). Subgroup analysis demonstrated an increased incidence of thrombocytopenia with abciximab (RR = 2.93; 95% CI 2.43–3.52) and tirofiban (RR = 2.79; 95% CI 1.17–6.63) only. Eptifibatide did not show any statistically significant increase in the risk of thrombocytopenia (RR = 1.05; 95% CI 0.86–1.29).

Our patient developed thrombocytopenia during the first 24 hours after his presentation to the hospital. The variations in the kinetics of the platelet count upon withdrawal of all antiaggregants seem to relate to TIT even though aspirin, ticagrelor, and enoxaparin are plausible etiologies for thrombocytopenia. Effectively, our patient being naïve to heparin would have developed HIT after five days. Moreover, aspirin and ticagrelor were never reported to cause such severe thrombocytopenia.

The time pattern of the TIT of our patient suggests the presence of naturally occurring antibodies that would destroy platelets coated with IIb/IIIa antagonists. Tirofiban may act as an antigen or may expose a platelet epitope that would be recognized by specific antibodies [8]. Our patient's thrombocytopenia was preceded by nonspecific allergic symptoms of palpitations, dyspnea, chills, and minor urticaria. Such reaction confirms the immune-related etiology of TIT. Our patient had refractory Crohn's disease which hypothetically increases the exposure of the humor immune system to colonic antigens which increases the formation of anti-GP IIb/IIIa antibodies [9]. The exposure of our patient to tirofiban, in the presence of anti-GP IIb/IIIa inhibitors, induced an allergic reaction that heralded the occurrence of thrombocytopenia. Therefore, we recommend monitoring of platelet counts in the subgroup of patients with autoimmune disease, particular inflammatory bowel disease, in case of tirofiban administration. These patients may benefit from prophylactic measures before administering GP IIb/IIIa inhibitors to prevent severe thrombocytopenia. Moreover, strict monitoring is required in patients who develop allergic reaction to tirofiban.
